# *catena*-Poly[[bis­(4-methyl­pyridine-κ*N*)cobalt(II)]-di-μ-dicyanamido-κ^2^ *N* ^1^:*N* ^5^]

**DOI:** 10.1107/S1600536812051252

**Published:** 2013-01-09

**Authors:** Wenjiang Huang, Jinfang Zhang, Chi Zhang

**Affiliations:** a Institute of Molecular Engineering and Advanced Materials, School of Chemical Engineering, Nanjing University of Science and Technology, 200 Xiaolingwei, Nanjing 210094, Jiangsu, People’s Republic of China; b Institute of Science and Technology, Jiangsu University, 301 Xuefu Road, Zhenjiang 212013, People’s Republic of China

## Abstract

Cobalt(II) nitrate hexa­hydrate and sodium dicyanamide self-assembled in dimethyl­formamide (DMF) and 4-methyl­pyridine solutions to form the title compound, [Co(C_2_N_3_)_2_(C_6_H_7_N)_2_]_*n*_. The Co^2+^ ion lies on an inversion center and adopts an octa­hedral coordination geometry in which four N atoms from four different dicyanamide ligands lie in the equatorial plane and two 4-methyl­pyridine N atoms occupy the axial positions. The Co^II^ atoms are connected by two bridging dicyanamide ligands, resulting in a chain parallel to the *c* axis. The chains are connected into a three-dimensional network by C—H⋯N hydrogen bonds.

## Related literature

The design and syntheses of metal-organic compounds has attracted great attention not only as a result of their intriguing architectures and topologies (Eddaoudi *et al.*, 2001 ▶), but also because of their potential applications (Banerjee *et al.*, 2008 ▶).
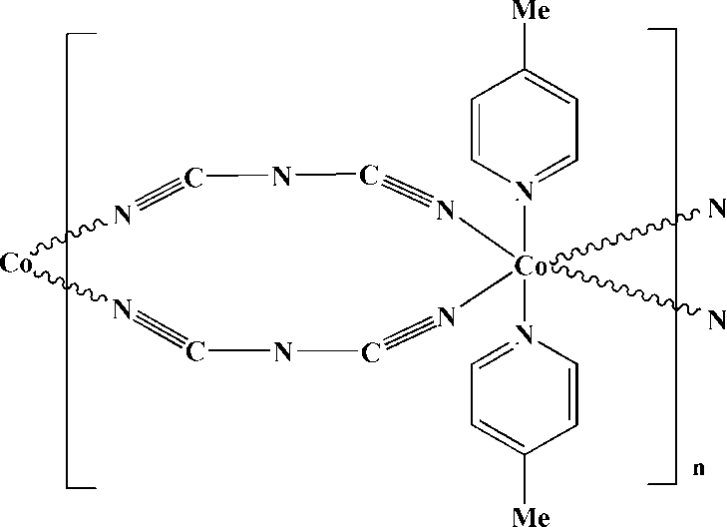


## Experimental

### 

#### Crystal data

[Co(C_2_N_3_)_2_(C_6_H_7_N)_2_]
*M* *_r_* = 377.28
Monoclinic, 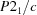

*a* = 9.3686 (19) Å
*b* = 13.080 (3) Å
*c* = 7.3048 (15) Å
β = 106.86 (3)°
*V* = 856.7 (3) Å^3^
*Z* = 2
Mo *K*α radiation
μ = 1.02 mm^−1^
*T* = 150 K
0.21 × 0.17 × 0.15 mm

#### Data collection

Rigaku Saturn724+ diffractometer
Absorption correction: multi-scan (*CrystalClear*; Rigaku, 2008 ▶) *T*_min_ = 0.815, *T*_max_ = 1.000
4994 measured reflections
1549 independent reflections
1419 reflections with *I* > 2σ(*I*)
*R*_int_ = 0.016

#### Refinement

*R*[*F* ^2^ > 2σ(*F* ^2^)] = 0.031
*wR*(*F* ^2^) = 0.085
*S* = 1.07
1549 reflections
115 parameters
H-atom parameters constrained
Δρ_max_ = 0.94 e Å^−3^
Δρ_min_ = −0.26 e Å^−3^

### 

Data collection: *CrystalClear* (Rigaku, 2008 ▶); cell refinement: *CrystalClear*; data reduction: *CrystalClear*; program(s) used to solve structure: *SHELXTL* (Sheldrick, 2008 ▶); program(s) used to refine structure: *SHELXTL*; molecular graphics: *SHELXTL*; software used to prepare material for publication: *SHELXTL*.

## Supplementary Material

Crystal structure: contains datablock(s) I, global. DOI: 10.1107/S1600536812051252/rz5034sup1.cif

Structure factors: contains datablock(s) I. DOI: 10.1107/S1600536812051252/rz5034Isup2.hkl

Additional supplementary materials:  crystallographic information; 3D view; checkCIF report

## Figures and Tables

**Table 1:** Hydrogen-bond geometry (Å, °)

*D*—H⋯*A*	*D*—H	H⋯*A*	*D*⋯*A*	*D*—H⋯*A*
C4—H4*A*⋯N3^i^	0.93	2.57	3.487 (3)	168

Symmetry code: (i) 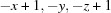.

**Fig. 1.**
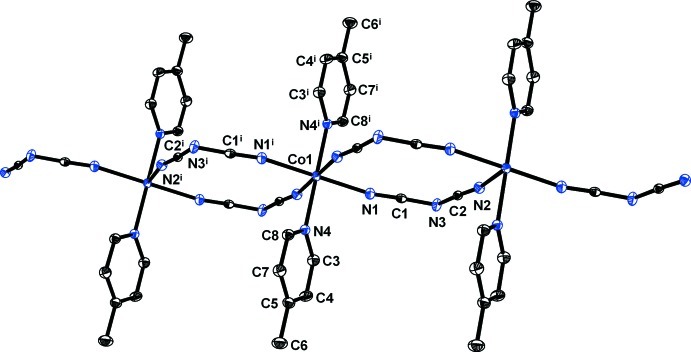
Portion of the polymeric chain of the title compound, with 30% probability displacement ellipsoids. All H atoms have been omitted. Symmetry code: (i) 2-x, -y, 1-z.

## supplementary crystallographic information

### Comment

The design and syntheses of metal-organic compounds have attracted great attention in recent years because of not only their intriguing architectures and topologies (Eddaoudi *et al.*, 2001) but also their potential applications (Banerjee *et al.*, 2008). The title compound {Co[N(CN)_2_]_2_(NC_6_H_7_)_2_}_n_ is constructed by the flexible dicyanamide bridging ligands through diffusion reaction.

As illustrated in Fig. 1, Co^2+^ ion lies on an inversion center and adopts an octahedral coordination geometry, where four N atoms from four different dicyanamide ligands lie in the equatorial plane and two 4-methylpyridine N atoms occupy the axial positions. The Co atoms are connected by two dicyanamide ligands, resulting in a neutral chain along the *c*-axis. In the crystal, the chains are linked by C—H···N hydrogen bonds (Table 1) into a three-dimensional network.

### Experimental

Co(NO_3_)_2_?6H_2_O (116.6 mg, 0.4 mmol) was added into 1 ml dmf with thorough stir for 5 minutes. After filtration, the purple filtrate was carefully laid on the surface with the solution of NaN(CN)_2_ (89.1 mg, 1 mmol) in 1 ml dmf, 1 ml 4-methylpyridine and 5 ml *i*-PrOH. Pink block crystals were obtained after two weeks.

### Refinement

H atoms were positioned geometrically and refined with riding model, with *U*_iso_ = 1.5*U*_eq_ and 1.2*U*_eq_ for methyl and pyridyl H atoms, respectively. The C—H bonds are 0.96 Å in methyl and 0.93 Å in pyridyl.

### Crystal data

**Table:**

[Co(C_2_N_3_)_2_(C_6_H_7_N)_2_]	*F*(000) = 386
*M**_r_* = 377.28	*D*_x_ = 1.463 Mg m^−^^3^
Monoclinic, *P*2_1_/*c*	Mo *K*α radiation, λ = 0.71073 Å
Hall symbol: -P 2ybc	Cell parameters from 3521 reflections
*a* = 9.3686 (19) Å	θ = 3.1–28.7°
*b* = 13.080 (3) Å	µ = 1.02 mm^−^^1^
*c* = 7.3048 (15) Å	*T* = 150 K
β = 106.86 (3)°	Block, pink
*V* = 856.7 (3) Å^3^	0.21 × 0.17 × 0.15 mm
*Z* = 2	

### Data collection

**Table:**

Rigaku Saturn724+ diffractometer	1549 independent reflections
Radiation source: fine-focus sealed tube	1419 reflections with *I* > 2σ(*I*)
Graphite monochromator	*R*_int_ = 0.016
ω scans	θ_max_ = 25.4°, θ_min_ = 3.3°
Absorption correction: multi-scan (*CrystalClear*; Rigaku, 2008)	*h* = −10→11
*T*_min_ = 0.815, *T*_max_ = 1.000	*k* = −12→15
4994 measured reflections	*l* = −8→8

### Refinement

**Table:**

Refinement on *F*^2^	Primary atom site location: structure-invariant direct methods
Least-squares matrix: full	Secondary atom site location: difference Fourier map
*R*[*F*^2^ > 2σ(*F*^2^)] = 0.031	Hydrogen site location: inferred from neighbouring sites
*wR*(*F*^2^) = 0.085	H-atom parameters constrained
*S* = 1.07	*w* = 1/[σ^2^(*F*_o_^2^) + (0.0483*P*)^2^ + 0.6117*P*] where *P* = (*F*_o_^2^ + 2*F*_c_^2^)/3
1549 reflections	(Δ/σ)_max_ < 0.001
115 parameters	Δρ_max_ = 0.94 e Å^−^^3^
0 restraints	Δρ_min_ = −0.26 e Å^−^^3^

### Special details

**Table:**

Geometry. All e.s.d.'s (except the e.s.d. in the dihedral angle between two l.s. planes) are estimated using the full covariance matrix. The cell e.s.d.'s are taken into account individually in the estimation of e.s.d.'s in distances, angles and torsion angles; correlations between e.s.d.'s in cell parameters are only used when they are defined by crystal symmetry. An approximate (isotropic) treatment of cell e.s.d.'s is used for estimating e.s.d.'s involving l.s. planes.
Refinement. Refinement of *F*^2^ against ALL reflections. The weighted *R*-factor *wR* and goodness of fit *S* are based on *F*^2^, conventional *R*-factors *R* are based on *F*, with *F* set to zero for negative *F*^2^. The threshold expression of *F*^2^ > σ(*F*^2^) is used only for calculating *R*-factors(gt) *etc*. and is not relevant to the choice of reflections for refinement. *R*-factors based on *F*^2^ are statistically about twice as large as those based on *F*, and *R*- factors based on ALL data will be even larger.

### Fractional atomic coordinates and isotropic or equivalent isotropic displacement parameters (Å^2^)

**Table:**

	*x*	*y*	*z*	*U*_iso_*/*U*_eq_	
Co1	1.0000	0.0000	0.5000	0.01854 (16)	
N1	0.86523 (19)	−0.07208 (14)	0.6510 (3)	0.0262 (4)	
N2	0.87457 (19)	−0.07142 (14)	1.2428 (2)	0.0249 (4)	
N3	0.76592 (19)	−0.12944 (15)	0.9113 (2)	0.0275 (4)	
N4	0.85008 (18)	0.12713 (13)	0.4394 (2)	0.0213 (4)	
C1	0.8228 (2)	−0.09665 (15)	0.7775 (3)	0.0203 (4)	
C2	0.8276 (2)	−0.09642 (15)	1.0853 (3)	0.0194 (4)	
C3	0.7023 (2)	0.11338 (17)	0.3732 (3)	0.0274 (5)	
H3B	0.6656	0.0469	0.3562	0.033*	
C4	0.6022 (2)	0.19299 (18)	0.3293 (3)	0.0315 (5)	
H4A	0.5006	0.1796	0.2828	0.038*	
C5	0.6525 (2)	0.29340 (17)	0.3544 (3)	0.0287 (5)	
C6	0.5461 (3)	0.3821 (2)	0.3074 (4)	0.0420 (6)	
H6A	0.4458	0.3570	0.2615	0.063*	
H6B	0.5682	0.4235	0.2105	0.063*	
H6C	0.5566	0.4225	0.4203	0.063*	
C7	0.8053 (2)	0.30713 (17)	0.4229 (3)	0.0301 (5)	
H7A	0.8448	0.3728	0.4420	0.036*	
C8	0.8989 (2)	0.22389 (16)	0.4628 (3)	0.0261 (5)	
H8A	1.0010	0.2353	0.5083	0.031*	

### Atomic displacement parameters (Å^2^)

**Table:**

	*U*^11^	*U*^22^	*U*^33^	*U*^12^	*U*^13^	*U*^23^
Co1	0.0207 (2)	0.0195 (2)	0.0153 (2)	−0.00038 (14)	0.00501 (16)	−0.00061 (14)
N1	0.0278 (9)	0.0278 (10)	0.0236 (9)	−0.0027 (7)	0.0086 (8)	0.0009 (8)
N2	0.0263 (9)	0.0267 (9)	0.0206 (10)	−0.0019 (7)	0.0053 (7)	−0.0036 (7)
N3	0.0242 (9)	0.0401 (11)	0.0181 (9)	−0.0131 (8)	0.0059 (7)	−0.0038 (8)
N4	0.0218 (8)	0.0219 (9)	0.0198 (9)	−0.0009 (7)	0.0053 (7)	−0.0008 (7)
C1	0.0169 (9)	0.0205 (10)	0.0201 (10)	−0.0007 (7)	0.0001 (8)	−0.0034 (8)
C2	0.0167 (9)	0.0191 (9)	0.0227 (11)	−0.0006 (7)	0.0064 (8)	0.0021 (8)
C3	0.0241 (10)	0.0258 (11)	0.0309 (12)	−0.0044 (8)	0.0056 (9)	−0.0032 (9)
C4	0.0193 (10)	0.0349 (12)	0.0386 (13)	−0.0010 (9)	0.0055 (9)	−0.0012 (10)
C5	0.0285 (11)	0.0283 (12)	0.0285 (11)	0.0055 (9)	0.0072 (9)	0.0017 (9)
C6	0.0370 (13)	0.0372 (14)	0.0511 (16)	0.0127 (11)	0.0118 (12)	0.0034 (12)
C7	0.0309 (11)	0.0213 (11)	0.0382 (13)	−0.0014 (9)	0.0102 (10)	−0.0010 (9)
C8	0.0217 (10)	0.0250 (11)	0.0306 (12)	−0.0024 (8)	0.0062 (9)	−0.0005 (9)

### Geometric parameters (Å, º)

**Table:**

Co1—N2^i^	2.1219 (18)	C3—C4	1.376 (3)
Co1—N2^ii^	2.1219 (18)	C3—H3B	0.9300
Co1—N1^iii^	2.1229 (18)	C4—C5	1.389 (3)
Co1—N1	2.1229 (18)	C4—H4A	0.9300
Co1—N4	2.1385 (17)	C5—C7	1.384 (3)
Co1—N4^iii^	2.1385 (17)	C5—C6	1.503 (3)
N1—C1	1.152 (3)	C6—H6A	0.9600
N2—C2	1.153 (3)	C6—H6B	0.9600
N2—Co1^iv^	2.1219 (18)	C6—H6C	0.9600
N3—C2	1.308 (3)	C7—C8	1.375 (3)
N3—C1	1.314 (3)	C7—H7A	0.9300
N4—C8	1.340 (3)	C8—H8A	0.9300
N4—C3	1.340 (3)		
			
N2^i^—Co1—N2^ii^	180.00 (7)	N2—C2—N3	175.1 (2)
N2^i^—Co1—N1^iii^	89.76 (7)	N4—C3—C4	123.1 (2)
N2^ii^—Co1—N1^iii^	90.24 (7)	N4—C3—H3B	118.5
N2^i^—Co1—N1	90.24 (7)	C4—C3—H3B	118.5
N2^ii^—Co1—N1	89.76 (7)	C3—C4—C5	120.2 (2)
N1^iii^—Co1—N1	180.00 (9)	C3—C4—H4A	119.9
N2^i^—Co1—N4	89.84 (7)	C5—C4—H4A	119.9
N2^ii^—Co1—N4	90.16 (7)	C7—C5—C4	116.5 (2)
N1^iii^—Co1—N4	90.06 (7)	C7—C5—C6	122.0 (2)
N1—Co1—N4	89.94 (7)	C4—C5—C6	121.5 (2)
N2^i^—Co1—N4^iii^	90.16 (7)	C5—C6—H6A	109.5
N2^ii^—Co1—N4^iii^	89.84 (7)	C5—C6—H6B	109.5
N1^iii^—Co1—N4^iii^	89.94 (7)	H6A—C6—H6B	109.5
N1—Co1—N4^iii^	90.06 (7)	C5—C6—H6C	109.5
N4—Co1—N4^iii^	180.0	H6A—C6—H6C	109.5
C1—N1—Co1	159.33 (16)	H6B—C6—H6C	109.5
C2—N2—Co1^iv^	163.96 (16)	C8—C7—C5	120.2 (2)
C2—N3—C1	117.09 (17)	C8—C7—H7A	119.9
C8—N4—C3	116.84 (18)	C5—C7—H7A	119.9
C8—N4—Co1	121.94 (13)	N4—C8—C7	123.2 (2)
C3—N4—Co1	121.22 (14)	N4—C8—H8A	118.4
N1—C1—N3	175.1 (2)	C7—C8—H8A	118.4

Symmetry codes: (i) *x*, *y*, *z*−1; (ii) −*x*+2, −*y*, −*z*+2; (iii) −*x*+2, −*y*, −*z*+1; (iv) *x*, *y*, *z*+1.

### Hydrogen-bond geometry (Å, º)

**Table:**

*D*—H···*A*	*D*—H	H···*A*	*D*···*A*	*D*—H···*A*
C4—H4*A*···N3^v^	0.93	2.57	3.487 (3)	168

Symmetry code: (v) −*x*+1, −*y*, −*z*+1.

## References

[bb1] Banerjee, R., Phan, A., Wang, B., Knobler, C., Furukawa, H., O’Keeffe, M. & Yaghi, O. M. (2008). *Science*, **319**, 939–943. 10.1126/science.1152516 18276887

[bb2] Eddaoudi, M., Moler, D. B., Li, H. L., Chen, B. L., Reineke, T. M., O’Keeffe, M. & Yaghi, O. M. (2001). *Acc. Chem. Res.* **34**, 319–330. 10.1021/ar000034b 11308306

[bb3] Rigaku (2008). *CrystalClear* Rigaku Corp., Tokyo, Japan.

[bb4] Sheldrick, G. M. (2008). *Acta Cryst.* A**64**, 112–122. 10.1107/S0108767307043930 18156677

